# The antibiotic vancomycin induces complexation and aggregation of gastrointestinal and submaxillary mucins

**DOI:** 10.1038/s41598-020-57776-3

**Published:** 2020-01-22

**Authors:** Vlad Dinu, Yudong Lu, Nicola Weston, Ryan Lithgo, Hayley Coupe, Guy Channell, Gary G. Adams, Amelia Torcello Gómez, Carlos Sabater, Alan Mackie, Christopher Parmenter, Ian Fisk, Mary K. Phillips-Jones, Stephen E. Harding

**Affiliations:** 1 National Centre for Macromolecular Hydrodynamics, School of Biosciences, University of Nottingham, Sutton Bonington, LE12 5RD UK; 2 Division of Food Science, School of Biosciences, Sutton Bonington, LE12 5RD UK; 3 School of Health Sciences, University of Nottingham, Nottingham, NG7 2HA UK; 4 School of Food Science & Nutrition, University of Leeds, Leeds, LS2 9JT UK; 5 Department of Bioactivity and Food Analysis, Institute of Food Science Research (CSIC-UAM), Nicolás Cabrera 9, 28049 Madrid, Spain; 6 Nottingham Nanoscale and Microscale Research Centre, University of Nottingham, University Park, Nottingham, NG7 2RD UK; 7 Kulturhistorisk Museum, Universitetet i Oslo, Postboks 6762, St. Olavs plass, 0130 Oslo, Norway

**Keywords:** Glycoconjugates, Bacterial infection

## Abstract

Vancomycin, a branched tricyclic glycosylated peptide antibiotic, is a last-line defence against serious infections caused by staphylococci, enterococci and other Gram-positive bacteria. Orally-administered vancomycin is the drug of choice to treat pseudomembranous enterocolitis in the gastrointestinal tract. However, the risk of vancomycin-resistant enterococcal infection or colonization is significantly associated with oral vancomycin. Using the powerful matrix-free assay of co-sedimentation analytical ultracentrifugation, reinforced by dynamic light scattering and environmental scanning electron microscopy, and with porcine mucin as the model mucin system, this is the first study to demonstrate strong interactions between vancomycin and gastric and intestinal mucins, resulting in very large aggregates and depletion of macromolecular mucin and occurring at concentrations relevant to oral dosing. In the case of another mucin which has a much lower degree of glycosylation (~60%) – bovine submaxillary mucin - a weaker but still demonstrable interaction is observed. Our demonstration - for the first time - of complexation/depletion interactions for model mucin systems with vancomycin provides the basis for further study on the implications of complexation on glycopeptide transit in humans, antibiotic bioavailability for target inhibition, *in situ* generation of resistance and future development strategies for absorption of the antibiotic across the mucus barrier.

## Introduction

**Figure 1:**
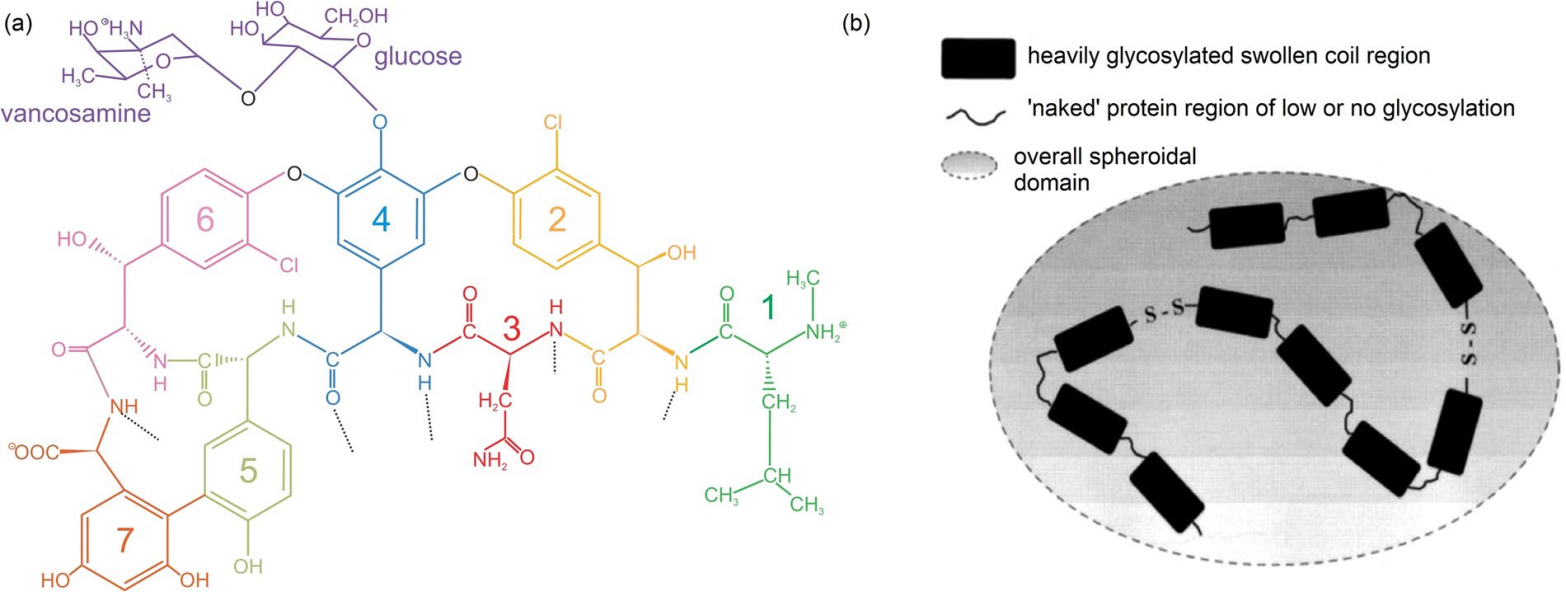
(**a**) Vancomycin structure (reproduced from Phillips-Jones *et al*.^4^ with permission from Nature Journals). The structure consists of a heptapeptide with an O-linked disaccharide. Molecular weight = 1449 g/mol. (**b**) Mucin structure. Based on a linear random coil model for colonic mucin (see ref. ^5^). Heavily glycosylated regions alternate with regions of low glycosylation, linked every 3-4 residues by disulphide bonds. The shadowed area shows the “effective” overall spheroidal volume of influence of this glycoprotein. The degree of glycosylation for most mucins of mammalian origin is ~80% although for submaxillary mucins it is lower (~60%). Molecular weights can be from 500,000–50 × 10^6^ g/mol depending on the source.

Vancomycin is a branched tricyclic glycosylated peptide antibiotic. In the clinic, it represents a last-line defence against infections caused by Gram-positive pathogenic bacteria. Isolated in 1956 and introduced into clinical practice in 1958, it acts by inhibiting cell wall synthesis in sensitive bacteria^1^. The largely hydrophilic molecule (see Fig. 1a) is able to form hydrogen bond interactions with the terminal d-alanyl-d-alanine moieties of the muramyl pentapeptide of the peptidoglycan. Under normal environments, the binding of vancomycin to d-Ala-d-Ala inhibits transglycosylase and transpeptidase activities during peptidoglycan growth, preventing the incorporation of new peptidoglycan into the expanding matrix, thereby leading to osmotic shock and cell lysis^2,3^.

Vancomycin was recently the subject of a detailed study using molecular hydrodynamics^4^. It was shown to form dimers (in common with other studies) and the reversibility and strength of the dimerization process in four different aqueous solvents (including a medically-used formulation) were studied using short-column sedimentation equilibrium in the analytical ultracentrifuge and model-independent SEDFIT-MSTAR analysis across a range of loading concentrations. The change in the weight average molar mass *M*_w_ with loading concentration was consistent with a monomer-dimer equilibrium. Overlap of data sets of point weight average molar masses *M*_w_(*r*) versus local concentration *c*(*r*) for different loading concentrations demonstrated a completely reversible equilibrium process. At the clinical infusion concentration of 5 mg/mL all glycopeptide was dimerized whilst at 19 µg/mL (a clinical target trough serum concentration), vancomycin was mainly monomeric ( < 20% dimerized)^4^.

Analysis of the variation of *M*_w_ with loading concentration revealed dissociation constants in the range 25–75 µM, commensurate with a relatively weak association. That study^4^, alongside companion studies^6,7^, also demonstrated a weak interaction with the A-type bacterial VanS histidine protein kinase involved in the activation of vancomycin resistance, at least in aqueous solution.

Despite its efficacy, vancomycin comes with potentially toxic side effects^8–11^, whether the antibiotic is administered intravenously or orally. The more intensive dosing regimens recommended by recent guidelines for intravenous vancomycin are associated with increasing reports of nephrotoxicity^12^; indeed, there is a long-standing, yet highly debated, association between vancomycin administration and nephrotoxicity^13,14^. Rare effects on hearing loss have also been reported^15,16^ through use of high peak serum concentrations of 49.2 µg/mL vancomycin^17^. Studies in recent years have rekindled interest in the best way to administer the antibiotic in the clinic to obtain the efficacious concentrations required to combat infections and yet minimise any toxic effects on patients^13,14^. To achieve this, many studies advocate a need to monitor serum concentrations of vancomycin with time^18–20^ and some suggest adjustment of loading doses (standardly 15 mg/kg, or 25–30 mg/kg also administered orally in the initial stages of therapy) according to patient disease state and body weight^20,21^, so that doses are individualised^22–24^. In general, in order to treat serious methicillin-resistant *Staphylococcus aureus* (MRSA) infections, the aim is to maintain vancomycin serum trough concentrations at 15–20 µg/mL^18,25,26^.

Vancomycin is also administered orally (typically at doses of 500 mg to 2 mg per day in 3-4 divided doses and resulting in stool concentrations of 1.4 mg/mL (ranging 0.5–2.0 mg/mL)^27^. It is used to treat pseudomembranous enterocolitis (PE), a disorder primarily caused by *Clostridium difficile* and occasionally by *S. aureus*^13,28^. Vancomycin is effective against both of these Gram-positive pathogens and is poorly absorbed from the gastrointestinal (GI) tract, thus making vancomycin the drug of choice for treatment of pseudomembranous enterocolitis (PE)^27^. However, orally-administered (and to a lesser extent intravenously-administered) vancomycin, results in development of vancomycin-resistant enterococci (VRE)^29,30^. The risk for VRE infection or colonization is significantly associated with oral vancomycin use^31–34^. It is possible that a component of the gastrointestinal tract contributes to selection of VRE through binding of vancomycin within the GI tract resulting in prolonged exposure periods for gut resident microorganisms such as the enterococci to acquire/ or express resistance. If delayed transit was the case, it is also noteworthy that vancomycin has significant and sometimes long-lasting inhibitory effects on the intestinal microbiota that is reported to be correlated with increased weight gain, asthma and diabetes in humans^9,35,36^.

Because of their protective function in the gastrointestinal tract, their exposed location for potential vancomycin interactions and their ability to provide a formidable permeability barrier with extensive binding properties, we investigate using model mucin systems whether gastrointestinal mucins are able to interact with vancomycin (a hydrophilic molecule that is weakly positively-charged at physiological pH). Any interactions could help explain the poor absorption of the antibiotic from the intestine and the glycopeptide impacts on the intestinal microbiota described above. The investigation involves the application of the powerful molecular hydrodynamic assay of co-sedimentation in the analytical ultracentrifuge to analyse interactions between different types of mucins and vancomycin. High rotor speeds (~45000 rpm, 130 000 g) are employed to sediment mucin (Fig. 1b) in solution while low rotor speeds (~3000 rpm) are used to sediment large supramolecular complexes of molecular weight > 10^8^Da. Our investigation reported here unequivocally demonstrates mucin-vancomycin interactions. We reinforce this demonstration of mucin complexation with dynamic (quasi-elastic) light scattering (DLS) measurements, a technique which is more sensitive to larger particles, and with environmental scanning electron microscopy.

*Model mucin systems*. Due to the difficulty in obtaining reproducible mucins from the gastrointestinal tract in sufficient quantity from human sources^37,38^ we follow the commonly accepted practice of using pig gastrointestinal mucins as our model mucin systems, both pig gastric mucin (PGM) and pig intestinal mucin (PIM)^39–43^. These appear to be the closest of the animal models to the gastrointestinal mucins for humans^39,44^. Although orally administered vancomycin is delivered through gelatin or other coated capsules – and hence with limited exposure to the mouth - another model mucin, bovine submaxillary mucin (BSM) is also considered because of its relatively low sugar content compared to the gastric and small intestinal mucins. The extension of our findings from these model mucins from animals to the case for humans is also considered.

## Results

Three concentrations of vancomycin were used in the present study. The rationale for choosing these is based on our previous findings^4^: at 0.125 mg/mL vancomycin is expected to be mostly monomeric, at 1.25 mg/mL (the typical concentration found in stools of patients given oral vancomycin) it is approximately 50% dimerized and at 12.5 mg/mL it is mostly dimeric. Concentrations of mucin were chosen to be low enough to be in the dilute region (non-molecular overlap): 0.5 mg/ml for PGM and PIM and 1.0 mg/ml for the smaller BSM.

### Analytical Ultracentrifugation (AUC) of solutions of pig gastric mucin (PGM) with vancomycin

**Figure 2:**
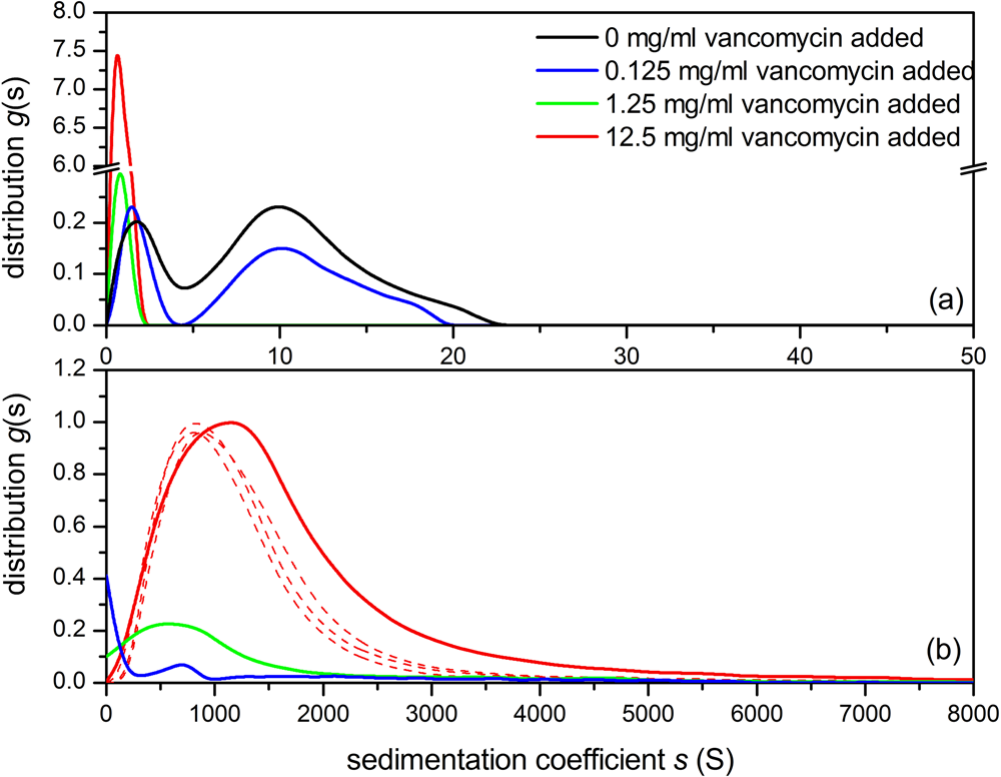
Sedimentation coefficient distribution of pig gastric mucin (PGM)/ vancomycin mixtures at different mixing ratio (**a**) at 45000 rpm (**b**) at 3000 rpm. 0.5 mg/mL PGM + 0.125 mg/mL (blue line), +1.25 mg/mL (dark green), +12.5 mg/mL (red) vancomycin. The 0.5 mg/mL PGM control is shown in black. The dashed lines represent repeats for 12.5 mg/mL vancomycin added.

The 45000 rpm plot (Fig. 2a), shows the 0.5 mg/mL pig gastric mucin control (no vancomycin added) showing two components with the main macromolecular mucin component of *s* values between 5 and 23 S and what happens to this as the vancomycin concentration is increased to 12.5 mg/mL. The amount of this component diminishes dramatically through complexation as the vancomycin concentration is increased. The 1.25 and 12.5 mg/mL additions indicate complete interaction of the mucin but this would be expected as the PGM concentration is much lower than the concentration of vancomycin. At 3000 rpm (Fig. 2b), the reverse is seen as the addition of vancomycin has produced large aggregates, (~1500 S), increasing as the vancomycin concentration is increased, leaving little macromolecular mucin behind as the vancomycin concentration is progressively raised. The extent of the depletion of mucin is given in Table 1.

**Table 1:** Proportion (%) of mucin lost (AUC) through complexation as a function of vancomycin added. Rotor speed 45000 rpm (130 000 g), 20.0 °C.

Mucin	Carbohydrate content (%)	Proportion of mucin lost (%) Vancomycin added (mg/ml)			
		0	0.125	1.25	12.5
PGM	80^a^	0	38	99	100
PIM	77^b^	0	23	100	100
BSM	60^c^	0	0	14	53

^a^Schömig *et al*.^45^; ^b^Mantle & Allen^46^; ^c^Tsuiki *et al*.^47^.

### AUC of solutions of pig intestinal mucin (PIM) with vancomycin

**Figure 3:**
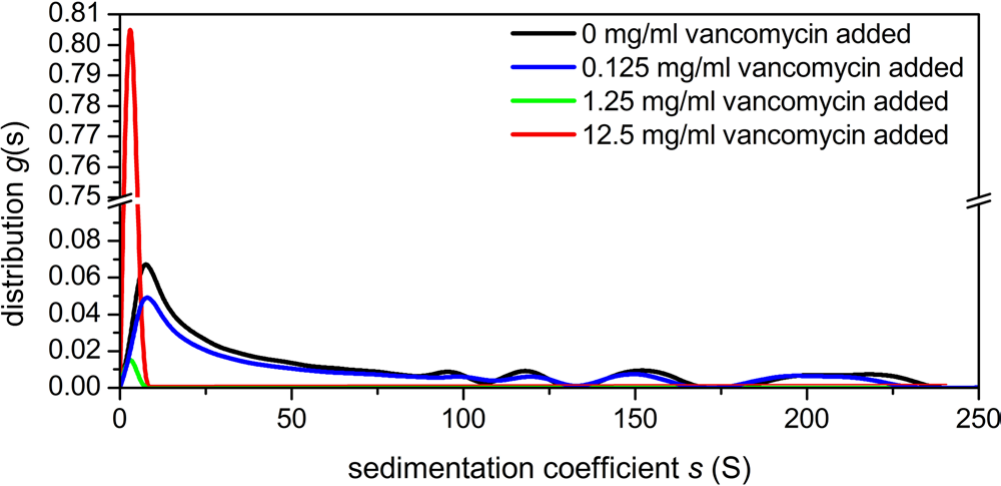
Sedimentation coefficient distribution of pig intestinal mucin (PIM) and vancomycin at differing mixing ratios. Rotor speed 45000 rpm. 0.5 mg/mL PIM + 0.125 mg/mL (blue line), +1.25 mg/mL (dark green), +12.5 mg/mL (red) vancomycin. The 0.5 mg/mL PIM control is shown in black.

At 0.5 mg/mL and 45000 rpm, the PIM control is seen with broad sedimentation coefficient distribution values of up to ~250 S. As with PGM, there is a progressive lowering in PIM concentration upon addition of increasing concentrations of vancomycin. At 0.125 mg/mL there is a small decrease while at 1.25 mg/mL and at 12.5 mg/mL the PIM disappears altogether, indicating complete interaction (Fig. 3). In common with observations with PGM, because of the lower mucin concentration relative to vancomycin, the excess vancomycin at 1.25 and 12.5 mg/mL concentrations is observed at very low sedimentation coefficient ( < 0.5 S). At 3000 rpm no trace is observed, and this is presumed to be due to the fast sedimentation of large vancomycin-mucin aggregates even at lower speed.

### AUC of solutions of bovine submaxillary mucin (BSM) with vancomycin

**Figure 4:**
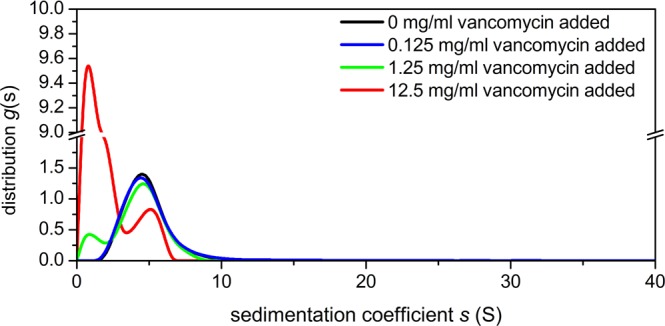
Sedimentation coefficient distribution of bovine submaxillary mucin (BSM) and vancomycin at differing mixing ratio. Rotor speed 45000 rpm. 1.0 mg/mL BSM + 0.125 mg/mL (blue line), +1.25 mg/mL (dark green), +12.5 mg/mL (red) vancomycin. The 1.0 mg/mL BSM control is shown in black.

Figure 4 shows the sedimentation coefficient distributions of the interaction of vancomycin with 1 mg/mL bovine submaxillary mucin of another model mucin, chosen because of its lower carbohydrate content (Table 1). Although there is a progressive loss of mucin concentration with the addition of vancomycin, the effects of the interaction were negligible at 0.125 and 1.25 mg/mL, respectively, as compared to pig gastric and intestinal mucins. There is however a significant loss in mucin concentration in the presence of 12.5 mg/mL vancomycin (Table 1). As with the previous experiments, the 1.25 and 12.5 mg/mL peaks at ~0.5 S represent changes in the presence of excess vancomycin.

### Dynamic Light Scattering (DLS)

**Figure 5:**
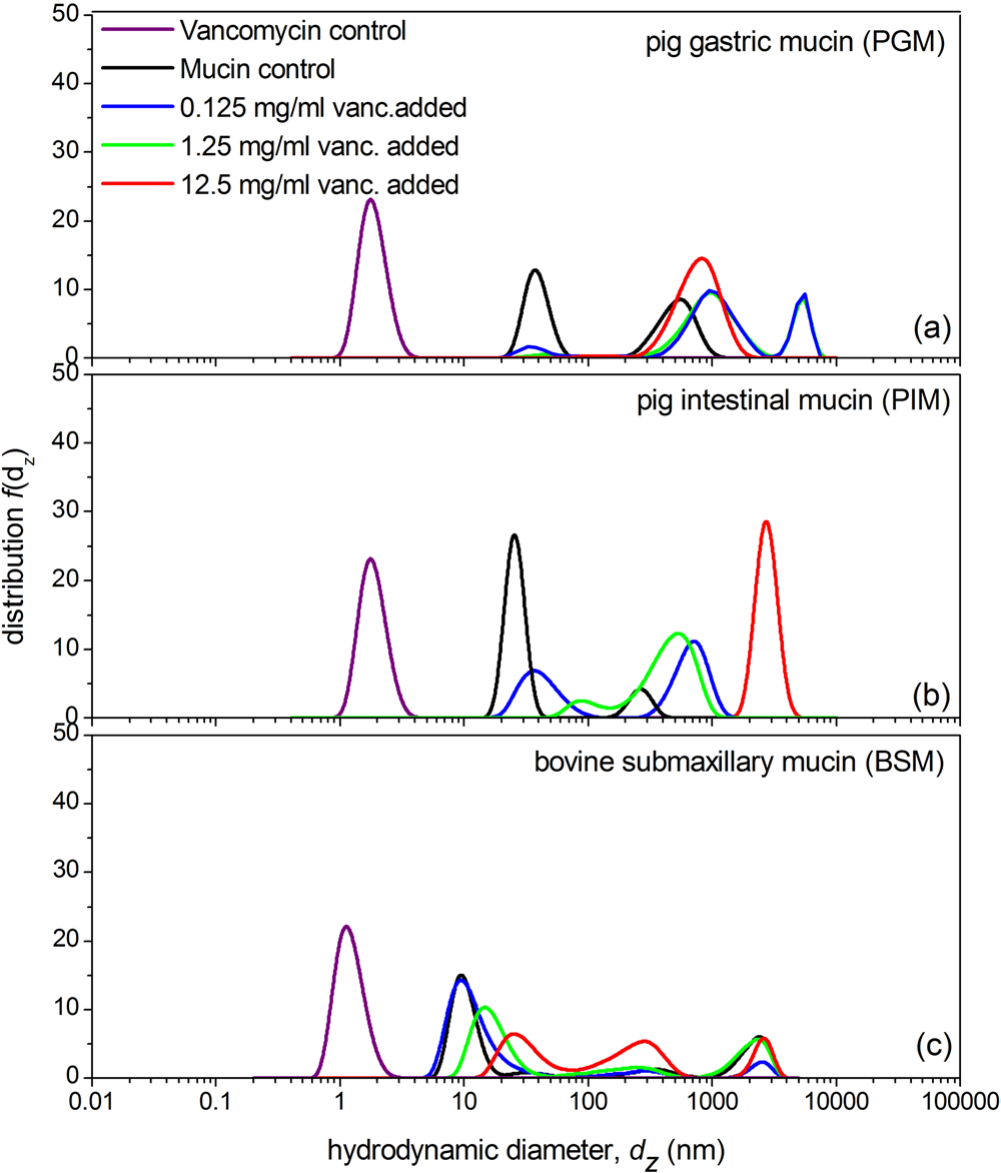
Distribution of z-average apparent hydrodynamic diameters, *d*_z,app_ from dynamic light scattering measurements on mucin and vancomycin at differing mixing ratios (**a**) 0.5 mg/mL PGM + 0.125 mg/mL (blue line), +1.25 mg/mL (dark green), +12.5 mg/mL (red). The 0.5 mg/mL PGM control is shown in black. (**b**) 0.5 mg/mL PIM + 0.125 mg/mL (blue line), +1.25 mg/mL (dark green), +12.5 mg/mL (red). The 0.5 mg/mL PIM control is shown in black and (**c**) 1.0 mg/mL BSM + 0.125 mg/mL (blue line), +1.25 mg/mL (dark green), +12.5 mg/mL (red). The 1.0 mg/mL BSM control is shown in black. Because free vancomycin scatters too weakly at the concentrations in the mixtures, for the vancomycin control (purple) a higher concentration of 50 mg/mL was used.

Further evidence of mucin-vancomycin complexation was sought using DLS and a Malvern Nanosizer-S instrument. The quasi-elastic or “dynamic” light scattering (DLS) data presented in Fig. 5 are shown in terms of Provencher (1992)^48^ - type of analysis of the distribution of apparent translational diffusion coefficients transformed via the Stokes-Einstein relation (Eq. 2) into apparent size (equivalent apparent hydrodynamic diameter). We use the “volume distribution” method. In a previous study by Malvern instruments^49^ the intensity, volume and number weighted distributions for a mixture of 60 and 200 nm latex spheres with a mass composition of 70% and 30% were compared: it was found the volume method gave an accurate reproduction of both the smaller and larger components, and much better than the other procedures so we follow that procedure here. However, values are apparent values as they are obtained at a fixed angle of 173° and we have followed the common practice of assuming overall particle sphericity (i.e. no contribution from possible rotational or other anisotropic effects). Although not as resolving^50^, and within the assumptions we have made, these results appear to confirm those from the analytical ultracentrifuge by showing the increased presence of large supramolecular complexes and depletion of the macromolecular mucin component upon addition of the vancomycin, particularly for the stomach and intestinal mucins which have a larger carbohydrate component: BSM, with lower glycosylation shows an interaction with lower depletion of the macromolecular mucin.

### Imaging of mucin-vancomycin complexes using environmental scanning electron microscopy (ESEM)

**Figure 6:**
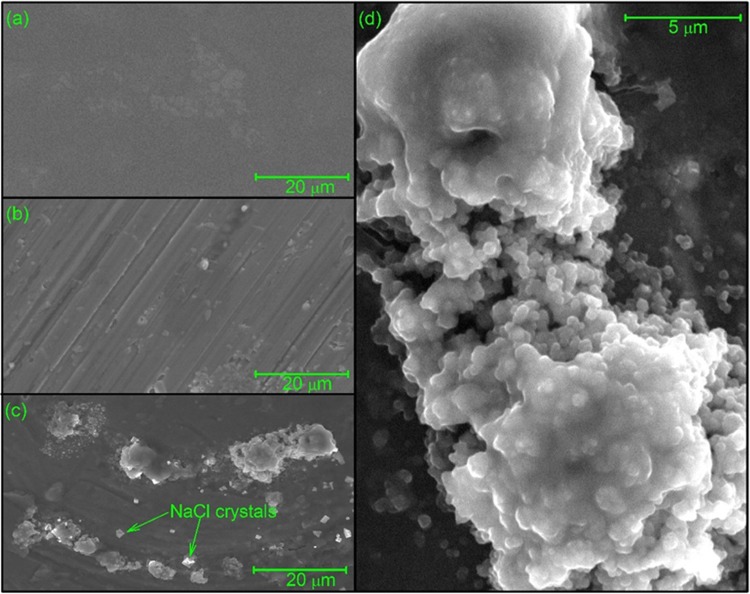
Environmental scanning electron micrographs of vancomycin (**a**), pig gastric mucins (**b**) and the complexes resulted from their interaction (**c,d**) suspended in 0.1 M PBS (pH 7.0). Aqueous samples were subjected to dehydration in the ESEM sample chamber at operating pressures ranging from ~4 to ~5 Torr. Small globular grains forming the supramolecular aggregate as shown in panel (d), a magnification of lower left panel (c).

**Figure 7:**
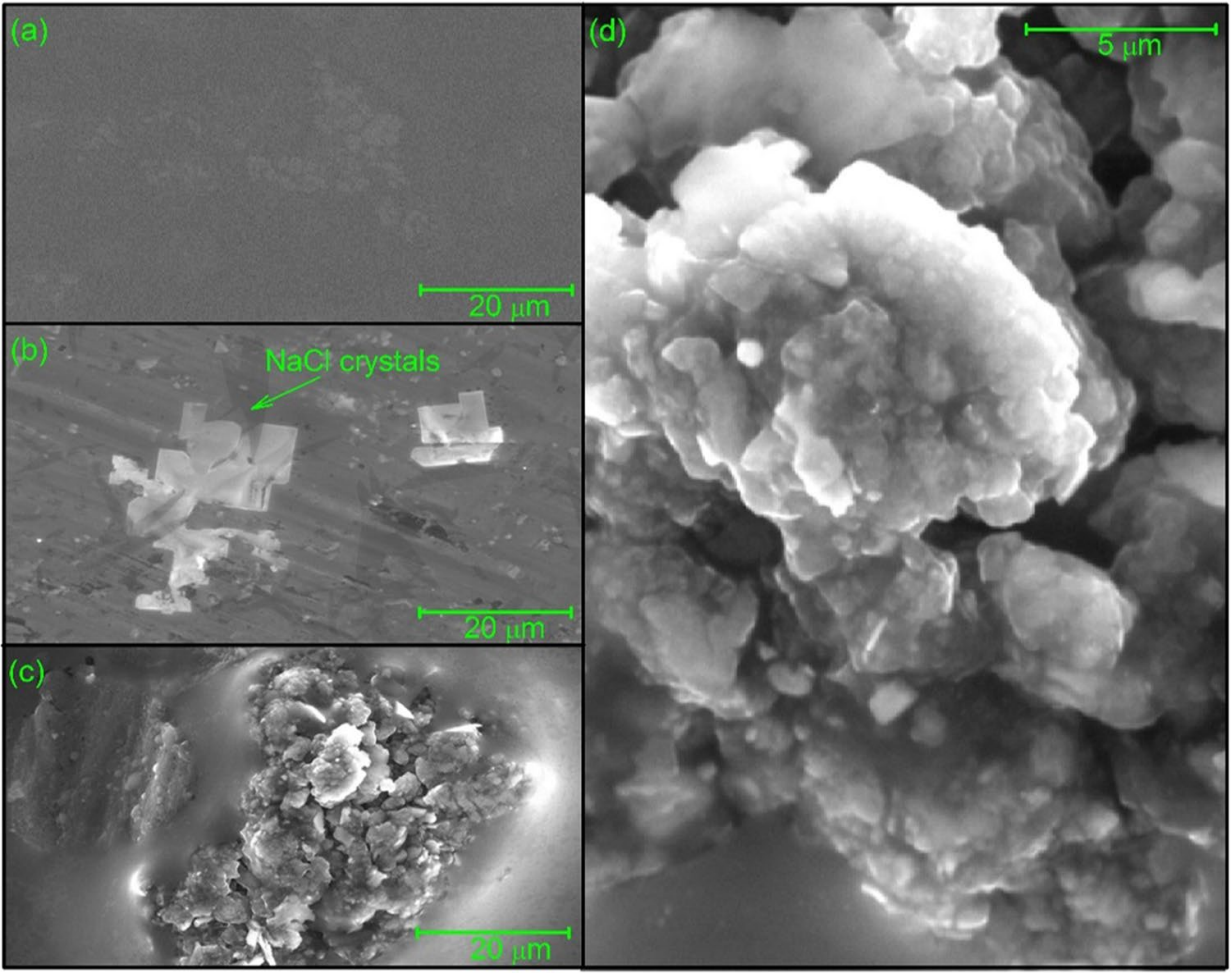
Environmental scanning electron micrographs of vancomycin (**a**), pig intestinal mucins (**b**) and the complexes resulted from their interaction with vancomycin (**c,d**) suspended in 0.1 M PBS (pH 7.0). Aqueous samples were subjected to dehydration in the ESEM sample chamber at operating pressures ranging from ~4 to ~5 Torr. Small globular grains forming the supramolecular aggregate as shown in panel (d), a magnification of lower left panel (c).

**Figure 8:**
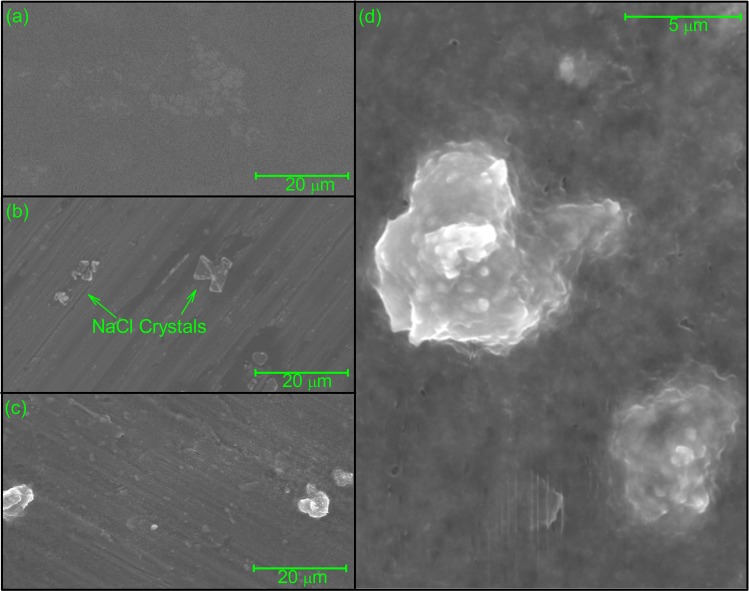
Environmental scanning electron micrographs of vancomycin (**a**), bovine submaxillary mucins (**b**) and the complexes resulted from the interaction with vancomycin (**c,d**) suspended in 0.1 M PBS (pH 7.0). Aqueous samples were subjected to dehydration in the ESEM sample chamber at operating pressures ranging from ~4 to ~5 Torr. Small globular grains forming the supramolecular aggregate as shown in panel (d), a magnification of lower left panel (c).

Electron microscopy and other imaging methods have been successfully applied in the past to investigate mucins and their complexation with mucoadhesives^51–56^. Here we use the technique of environmental scanning electron microscopy (ESEM)^57^ to visualise the large supramolecular complexes resulting from the interactions of vancomycin with PGM, PIM and BSM under controlled dehydration in the ESEM sample chamber. Mucin-vancomycin mixtures, or vancomycin alone or mucin alone controls were observed under an operating pressure of ~4 to 5 Torr (Figs. 6–8).

Although the technique of ESEM is useful for the visualisation of a range of sample sizes from ~10 nm to large cells and tissues^58–60^, so far (and to the best of our knowledge) there has been limited application of ESEM for the study of antibiotic complexation. In our study, the samples were analysed under the same chemical conditions as previously used for the AUC and DLS experiments described above, with samples (prior to drying) in 0.1 M PBS. The conditions employed resulted in the formation of salt crystals interspersed between the molecules.

For the PGM system, at a constant magnification (Fig. 6a–c), few (if any) visible particles were evident for the vancomycin-only control, apart from the small air bubbles formed during drying (Fig. 6a). The PGM mucin-only control sample showed formation of well-separated smooth elongated structures ( < 1 µm) (Fig. 6b). By contrast, the mucin-vancomycin mixture showed very large aggregates (Fig. 6c), consistent with our hydrodynamic analysis. Closer examination of these aggregates showed that they were composed of spherical grains (~0.2 µm) that were not observed in either of the controls, and which appeared to clump together to form the large aggregate structures shown (Fig. 6d). Very similar behaviour was seen for the PIM system with large aggregation in the presence of vancomycin but nothing in the absence apart from salt crystals (Fig. 7). Similar behaviour was also seen for the bovine submaxillary mucin although complexes were generally smaller (Fig. 8).

Therefore, the ESEM analysis undertaken here appears to reinforce the hydrodynamic data that vancomycin induces mucin aggregation. The exact mechanism is currently under investigation but electrostatic binding of the charged amino groups (possessing an overall positive charge of ~+0.67 at neutral pH) present on the vancomycin to the sialic acid residues present in the mucin may at least be a contributory factor. i.e. this may involve “bridging” electrostatic forces as have been well described for other systems^61–64^. It is worth noting that in the stomach the pH (1.5–3.5) is ~ the pKa of sialic acid (2.5), whereas the pH of the intestine is ~ 6.0–7.4 and that of the mouth ~7.5. The effect of pH on the interactions and also the influence of bile salts – with the appropriate vancomycin and mucin controls (see, for example refs. ^65,66^) would be a fruitful source of further investigation.

## Discussion

If we make the assumption that our observations for model mucin systems – based on the commonly used pig mucin model - can be extended to human mucin systems, vancomycin-mucin interactions may provide one explanation for the well documented poor absorption characteristics of orally-administered vancomycin^13,27^. The large mucin glycoproteins, together with salts, lipids and other proteins that make up mucus, provide a strong selective barrier for passage of only low molecular weight components such as mono-, di- or tri-peptides^67,68^ and hydrophilic, net neutral charge particles that can permeate the mucin mesh size of ~100 nm^69,70^; monomeric vancomycin is a large glycopeptide with a molar mass of ~1500 Da.

The poor absorption of vancomycin is presumably attributable in part to its relatively larger mass and also its overall positive charge at^71^ pH < ~8.5 which prevents it from penetrating the mucus layer by binding and aggregation events. Our current findings indicate that the binding of vancomycin to mucin itself would provide an additional and effective mechanism for its exclusion from intestinal and gastric absorption when the antibiotic is administered orally. It seems possible to conclude from our studies that mucin binding by vancomycin occurs whether it is in mainly dimeric or monomeric forms, as suggested by the consistent binding observed when the antibiotic is mainly monomeric (at 0.125 mg/mL), approximately 50% dimerized (at 1.25 mg/mL, the typical stool concentration) and at the higher 12.5 mg/mL at which the antibiotic is mostly dimeric^4^, though further studies are required to verify this.

However, it should also be borne in mind that, for most glycopeptide antibiotics studied to date^72–74^ including vancomycin^66^, it has been shown that binding to depsipeptide targets is accompanied by formation of asymmetric, back-to-back homodimers of the glycopeptide in aqueous solution and that this is mediated by sugar-sugar recognition^75,76^. Although glycopeptide antibiotics are known to bind or interact with protein targets such as d-alanyl-d-alanine and VanS^4,6^, precipitation with another glycoprotein, heparin, has also been reported previously. However, this only appears to occur at relatively high concentrations of both components such as those used in intravenous lines (vancomycin at 1–5 mg/mL and heparin at 1–1000 Units/mL)^77–80^.

Administration of vancomycin, whether orally or intravenously, has been reported to result in development of vancomycin-resistant enterococci (VRE) populations^29,30^. However, it is the oral administration that appears to be the most strongly associated with increased risk of VRE infection or colonization^30–33^. Although the emergence of VRE as nosocomial pathogens is thought to occur from VRE reservoirs built up in the antibiotic-perturbed GI tract following ingestion from the hospital environment^81,82^, it is also possible that some contribution to the emergence of VRE populations (including in healthy, community-based individuals who carry low-level VRE) may be made by the possible prolonged period of time that vancomycin remains in the GI tract resulting from mucin binding, assuming that aggregates are not rapidly cleared from the GI tract and that the antibiotic remains exposed within the aggregated state.

Mucin binding to vancomycin may provide increased exposure for target pathogens and for resident mucin-bound enterococci and other microorganisms to the antibiotic, resulting in increased activation of resistance and selection for VRE populations. If confirmed, this finding may be of significance not only for oral administration strategies but also for adjunctive intracolonic vancomycin (ICV) therapy strategies which are also used to treat pseudomembranous colitis^83^.

To conclude, there is evidence of strong complexation between vancomycin and model mucins from different parts of the GI tract, as shown by analytical ultracentrifugation (Figs. 2–4), DLS (Fig. 5) and further confirmed by ESEM (Figs. 6–8). The strongest interactions - with very large complexes being formed (Figs. 2 and 3) - appear to be associated with mucus originating from the stomach and small intestine, compared with the mouth. The lower degree of association of the latter (Fig. 4, Table 1) may be connected with the lower degree of glycosylation of submaxillary mucins. Our study suggests that vancomycin may interact with the carbohydrate region of mucins, although interactions with the protein component of mucins cannot be excluded. In that regard it would be interesting to explore further the role of the glycosylation of mucins and to O-deglycosylate the proteins (or find sources of non-glycosylated recombinant mucins) and perform similar studies with vancomycin – provided the deglycosylated mucins are sufficiently soluble.

Finally it should be stressed that although pig gastrointestinal mucins are considered a good model for human mucins the extension of our conclusions to the case for humans is still only provisional. Nonetheless our demonstration - for the first time - of complexation/depletion interactions for model mucin systems with vancomycin provides the basis for further study on how orally administered vancomycin might relate to glycopeptide transit in humans^67^.

## Methods

### Vancomycin

Vancomycin hydrochloride was obtained from Sigma-Aldrich, United Kingdom. A partial specific volume ῡ of 0.67 mL g^−1^ was used^4,84^. Solution concentrations of vancomycin *c* (g/mL) were determined densimetrically from the relation:

1 $$c=(\rho -{\rho }_{o})/(1-\bar{\upsilon }{\rho }_{o})$$

where ρ and ρ_o_ are the solution and solvent densities respectively, measured on an Anton-Paar (Graz, Austria) digital density meter. This is simply a re-arrangement of the well-established relation^85^ of Kratky and co-workers for the partial specific volume measured at a single solute concentration.

### Pig gastric mucin, PGM

A 5 mg/mL stock solution of pig gastric mucin was prepared by dissolving 50 mg of porcine stomach mucin (Sigma-Aldrich, catalogue no. M1778, type III) in 10 mL of phosphate-buffered saline (PBS) buffer, pH 7.0, adjusted to an ionic strength I = 0.1 M by the addition of NaCl (Life Technologies Australia Pty Ltd., Invitrogen division, catalogue no. 14190250) according to Green^86^.

### Pig intestinal mucin, PIM

This was prepared as described previously^87^. Fresh porcine small intestine obtained from a local slaughterhouse was rinsed with 67 mM phosphate buffer (pH 6.7) containing 0.02% w/v sodium azide and a mix of protease inhibitors (Roche Diagnostics GmbH, Mannheim, Germany; 1 tablet per 50 mL buffer) to remove debris. Mucus was removed by a gentle scraping of the epithelial surface of the jejunum segment of the intestine with a plastic scraper (Corning, NY, USA). Extraneous debris (such as dead epithelial cells) was removed by extracting the mucus overnight at 18–22 °C with gentle (30 rpm) stirring in 7 volumes of extraction buffer (10 mM sodium phosphate, pH 6.5, containing 4 M guanidine hydrochloride, 5 mM EDTA, 5 mM N-ethylmaleimide and 0.02% w/v sodium azide). Precipitated material was collected by centrifugation for 30 min at 22000 × g (10.0 °C) and re-extracted in the same way with 10 volumes of extraction buffer, followed by centrifugation for 30 min at 22000 × g (10 °C). The insoluble precipitate (crude mucin) was collected and stored at −80 °C. The crude mucin was diluted with 10 mM sodium phosphate buffer (pH 6.5) containing 6 M guanidine hydrochloride and adjusted to a density of 1.4 g/mL with CsCl and centrifuged (55000 rpm at 10.0 °C for 62 h).

Aliquots of 0.5 mL were sampled, the absorption at 280 nm was measured, and 2 μL of each fraction was spotted and stained with Alcian blue. UV- and Alcian blue-positive aliquots were pooled and diluted in extraction buffer lacking guanidine hydrochloride (final guanidine hydrochloride concentration 0.5 M), adjusted in density to 1.41 g/mL with CsCl and centrifuged again (50000 rpm at 10.0 °C for 96 h). One-millilitre aliquots were sampled, measured at 280 nm and stained with Alcian blue. The fraction at densities of 1.55–1.60 g/mL was strongly Alcian blue-positive and had very weak absorption at 280 nm, identifying it as the mucin fraction. This fraction was then dialysed against phosphate-buffered saline (PBS; 10000 g/mol cut-off tubing) and stored at 4 °C before use. All solutions were made up in PBS to a final ionic strength of 0.1 M and pH 7.0 to a stock concentration of ~0.7 mg/mL.

### Bovine submaxillary mucin, BSM

A 5 mg/mL stock solution was prepared by dissolving 50 mg of BSM (Sigma-Aldrich, catalog no. M3895, type I-S) in 10 ml of phosphate-buffered saline (PBS) buffer, pH7.0, adjusted to an ionic strength I = 0.1 M as described previously^86^.

### Sedimentation velocity in the Analytical Ultracentrifuge (AUC)

Experiments were performed at 20.0 °C using the Optima XL-I analytical ultracentrifuge (Beckman, Palo Alto, USA) equipped with Rayleigh interference optics, as described previously^88^. Sample volumes of 395 μl (or 405 μl for reference solvent), were injected into the 12 mm double sector epoxy cells with sapphire windows and run at 45000 rpm. Scans were taken at 2 minute intervals. The interference system produced data derived by recording changes in concentration (in fringe units) versus radial displacement. The data was analysed in SEDFIT using the least squares, ls-g*(*s*) processing method - *g*(*s*) by generating sedimentation coefficient distributions, *s*_20,w_ (in Svedberg units, S = 10^−13^sec) normalised to standard conditions (viscosity, density of solvent at 20.0 °C)^4,6^. The following mixing ratios were used (Figs. 2–3): 0.5 mg/mL mucin + 0.125 mg/mL vancomycin (blue line), +1.25 mg/mL (dark green), +12.5 mg/mL (red) vancomycin. The 0.5 mg/mL mucin controls are shown in black. For BSM, (Fig. 4) we used a higher concentration of ~1 mg/mL to compensate for its lower molecular weight and lower non-ideality for a given concentration.

### Dynamic light scattering

Dynamic or quasi-elastic light scattering (DLS or QLS) measurements were made on the fixed scattering angle Zetasizer Nano-S system (Malvern Instruments Ltd., Malvern, UK)^50,89^, equipped with a 4 mW He-Ne laser at a wavelength of 632.8 nm. Samples in solution were measured in a quartz cuvette at 20.0 °C. A scattering angle of 173° was used, and collected in manual mode, requiring a measurement duration of 90 seconds. The resulting data were analysed using the “DTS (Version 4.2)” software (Malvern Instruments Ltd., Malvern, UK), providing a volume distribution of translational diffusion coefficients based on the CONTIN program of Provencher (1992)^48^. The volume distribution was followed^49^. The viscosity of the buffer used was calculated using a solvent builder interface and takes the effects of buffer salts into account.

The apparent z-average apparent hydrodynamic diameters *d*_*z,app*_ (nm), were evaluated from the z-average apparent translational diffusion coefficients *D*_z_,_trans,app_ = by the Stokes-Einstein equation (see e.g., Harding *et al*.)^89^:

2 $${d}_{z,app}={k}_{B}T/\{3\pi \eta {D}_{z,trans,app}\}$$

where *k*_*B*_ is the Boltzmann constant, *T* is absolute temperature and η is the viscosity of the medium. The following assumptions were made (i) the solutions were sufficiently dilute that non-ideality effects were not significant – i.e. an extrapolation to zero concentration was not necessary. This is reasonable as the non-ideality due to the low concentration of mucin and small size of vancomycin, and also for translational diffusion the two main contributory factors to non-ideality – the hydrodynamic and thermodynamic terms - compensate for each other and can even cancel each other out^90,91^. (ii) the particles (vancomycin, mucin and complex) were quasi-spheroidal and not asymmetric so there was no angular dependence of the measured *D*_z,trans_ values on anisotropic rotational diffusion effects – i.e. an extrapolation to zero angle was not necessary^92^.

The following mixing ratios were used (Fig. 5): (a) 0.5 mg/mL PGM + 0.125 mg/mL vancomycin (blue line), +1.25 mg/mL (dark green), +12.5 mg/mL (red). The 0.5 mg/mL PGM control is shown in black. (b) 0.5 mg/mL PIM + 0.125 mg/mL (blue line), +1.25 mg/mL (dark green), +12.5 mg/mL (red). The 0.5 mg/mL PIM control is shown in black and (c) 1.0 mg/mL BSM + 0.125 mg/mL (blue line), +1.25 mg/mL (dark green), +12.5 mg/mL (red). The 1.0 mg/mL BSM control is shown in black. Because free vancomycin scatters too weakly at the concentrations in the mixtures, for the vancomycin control (purple) a higher concentration of 50 mg/mL was used.

### Environmental Scanning Electron Microscopy (ESEM) analysis

Vancomycin and mucin samples were analysed using a Thermofisher Scientific (Waltham, USA) FEI Quanta 650 ESEM. Samples were cooled to 2.0 °C by means of a Peltier cooling stage, and the pressure of water vapour in the chamber was adjusted to maintain a relative humidity of between 80 to 90%, or for the mucin control between 50 to 60%. An accelerating voltage of 15 kV was used for all samples. The following mixing ratios were used: 0.5 mg/mL mucin control, 12.5 mg/mL vancomycin control and 0.5 mg/mL + 12.5 mg/mL mixture.
